# Impact of myocardial perfusion abnormalities on clinical outcomes in patients treated with percutaneous coronary intervention for chronic total occlusions

**DOI:** 10.1093/ehjimp/qyaf137

**Published:** 2026-01-08

**Authors:** Jesper Boes Henningsen, Marc Meller Søndergaard, Steen Hyldgaard Jørgensen, Jacob Hartmann Søby, Morten Böttcher, Laust Dupont Rasmussen, Evald Høj Christiansen, Emil Nielsen Holck, Lisette Okkels Jensen, Karsten Tange Veien, Kirsten Bouchelouche, Christian Torp Pedersen, Kristian Hay Kragholm, Ashkan Eftekhari

**Affiliations:** Department of Cardiology, Aalborg University Hospital, Hobrovej 18-20, Aalborg 9000, Denmark; Department of Cardiology, Aalborg University Hospital, Hobrovej 18-20, Aalborg 9000, Denmark; Department of Cardiology, Aalborg University Hospital, Hobrovej 18-20, Aalborg 9000, Denmark; Department of Cardiology, Gødstrup Hospital, University Clinic for Cardiovascular Research, Herning, Denmark; Department of Cardiology, Gødstrup Hospital, University Clinic for Cardiovascular Research, Herning, Denmark; Department of Cardiology, Aalborg University Hospital, Hobrovej 18-20, Aalborg 9000, Denmark; Department of Cardiology, Aarhus University Hospital, Aarhus, Denmark; Department of Internal Medicine and Cardiology, Horsens Regional Hospital, Horsens, Denmark; Department of Cardiology, Odense University Hospital, Odense, Denmark; Department of Cardiology, Odense University Hospital, Odense, Denmark; Department of Nuclear Medicine, Aarhus University Hospital, Aarhus, Denmark; Department of Cardiology and Clinical Investigation, North Zealand Hospital, Hillerød, Denmark; Department of Cardiology, Aalborg University Hospital, Hobrovej 18-20, Aalborg 9000, Denmark; Department of Cardiology, Aalborg University Hospital, Hobrovej 18-20, Aalborg 9000, Denmark

**Keywords:** myocardial perfusion, chronic total occlusions, coronary artery disease, angina pectoris, percutaneous coronary intervention, ischaemia

## Abstract

**Introduction:**

Myocardial perfusion imaging (MPI) is used to evaluate ischaemia in patients with chronic total occlusion (CTO), but its prognostic implications following percutaneous coronary intervention (PCI) of CTO remain uncertain.

**Purpose:**

To evaluate outcomes in patients treated with CTO-PCI stratified by moderate–severe ischaemia on MPI prior to intervention.

**Methods and results:**

Patients from the Western Danish Heart Registry assessed by nuclear MPI and subsequently treated with CTO-PCI ≤ 6 months were included. Moderate–severe ischaemia was defined as ≥10% left ventricle involvement. Primary endpoints were all-cause mortality and a composite of major adverse cardio- and cerebrovascular events [MACCE; cardiovascular death, myocardial infarction (MI), stroke, and hospitalization for heart failure (HF) or angina pectoris]. Secondary endpoints included the individual MACCE components. Outcomes were compared between patients with and without moderate–severe ischaemia using multivariable Cox regression and competing risk regression at 90-day and 5-year follow-ups. Among 319 patients, 208 (65.2%) had moderate–severe ischaemia. All-cause mortality was similar between patients with and without moderate–severe ischaemia [adjusted hazard ratio (aHR) 1.12, 95% confidence interval (CI): 0.52–2.43], *P* = 0.77). The estimated risk of MACCE was comparable between groups at 90 days [aHR 0.76 (0.38–1.55), *P* = 0.46] and 5 years [aHR 0.74 (0.45–1.20), *P* = 0.22]. No difference was found in MI [5 years: aHR 0.76 (0.26–2.22), *P* = 0.61] or hospitalization for HF [90 days: aHR 0.44 (0.16–1.21), *P* = 0.11]; 5 years: aHR 0.62 (0.30–1.30), *P* = 0.21]. Hospitalization for angina was similar at 90 days [aHR 0.75 (0.26–2.16), *P* = 0.60], but a decreased 5-year risk was observed in patients with moderate–severe ischaemia [aHR 0.46 (0.23–0.91), *P* = 0.026].

**Conclusion:**

Moderate–severe ischaemia on nuclear MPI was not associated with differences in mortality or MACCE after CTO-PCI but was associated with a lower long-term risk of angina hospitalization.

## Graphical Abstract

**Graphical Abstract:**
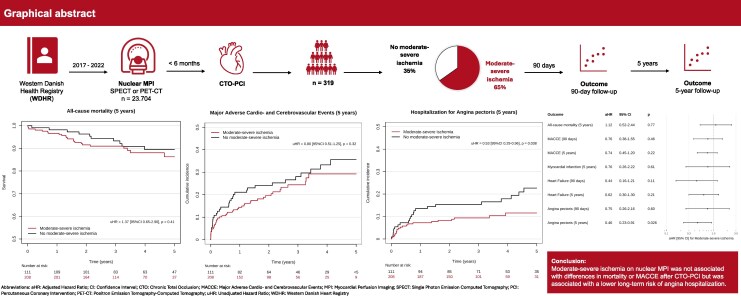

## Introduction

Chronic total occlusion (CTO) is defined as a complete occlusion of a coronary artery with no measurable blood flow (thrombolysis in myocardial infarction flow 0) for at least 3 months.^1^ CTO lesions are common, occurring in approximately 13–30% of patients with clinically significant coronary artery disease (CAD).^2–4^ Patients with CTO have an increased risk of ventricular arrythmia,^5^ myocardial infarction (MI) and mortality.^6^ Moreover, percutaneous coronary intervention (PCI) of CTO lesions is technically demanding and associated with a higher risk of complications, thus representing a substantial healthcare problem.^7,8^

When compared with optimal medical treatment, CTO-PCI has been associated with a reduced risk of mortality, MI, and target vessel revascularization in observational studies.^9–11^ However, randomized clinical trials failed to demonstrate a prognostic benefit, although two trials indicated better symptom control and improved quality of life.^12–14^ Consequently, current guidelines from the American Heart Association (AHA) and a clinical consensus statement from the European Society of Cardiology (ESC) recommend ischaemic symptom improvement as the primary indication for CTO-PCI.^1,15^ As CTO-PCI techniques have been refined, procedure-related success rates are markedly increasing and complication rates are steadily declining.^16^ Successful CTO-PCI as compared to non-successful CTO-PCI has been linked to decreased mortality and major adverse cardiovascular or cerebral events, as well as decreased angina frequency and need for subsequent coronary artery bypass graft (CABG).^17–19^

Non-invasive myocardial perfusion imaging (MPI), including single photon emission computed tomography (SPECT) or positron emission tomography (PET), plays a valuable role in the pre-procedural evaluation of patients with CTOs.^20,21^ Moderate–severe ischaemia, defined as an area of ischaemia ≥10% of the left ventricular myocardium, is recommended by the ESC and AHA as the threshold for identifying patients at increased risk of future adverse events.^22–24^ In patients with stable CAD, Shaw *et al*. demonstrated a greater reduction in ischaemia on MPI when adding PCI to optimal medical treatment and a subsequently lower risk of adverse events among patients with reduced ischaemia. However, the recent International Study of Comparative Health Effectiveness with Medical and Invasive Approaches (ISCHEMIA) trial did not show superiority of an initial invasive strategy compared with an initial conservative strategy in patients with moderate–severe ischaemia.^25,26^ Overall, the prognostic implications of myocardial ischaemia in patients with CTO lesions undergoing PCI are not fully understood.

This study aims to evaluate outcomes in patients undergoing CTO-PCI with and without evidence of moderate–severe myocardial ischaemia on MPI using a large-scale clinical database.

## Method

### Design and study population

This study is an observational, registry-based cohort study utilizing data from the Western Denmark Heart Registry (WDHR), a validated clinical and treatment database prospectively collecting patient and procedural data from all diagnostic and interventional procedures conducted at the three main intervention centres in Western Denmark: Aarhus University Hospital, Odense University Hospital, and Aalborg University Hospital and nine referring regional hospitals.^27^ The data regarding nuclear-based MPI procedures have recently been validated and showed high completeness and validity, supporting its use for cardiac research.^28^

**Figure 1:**
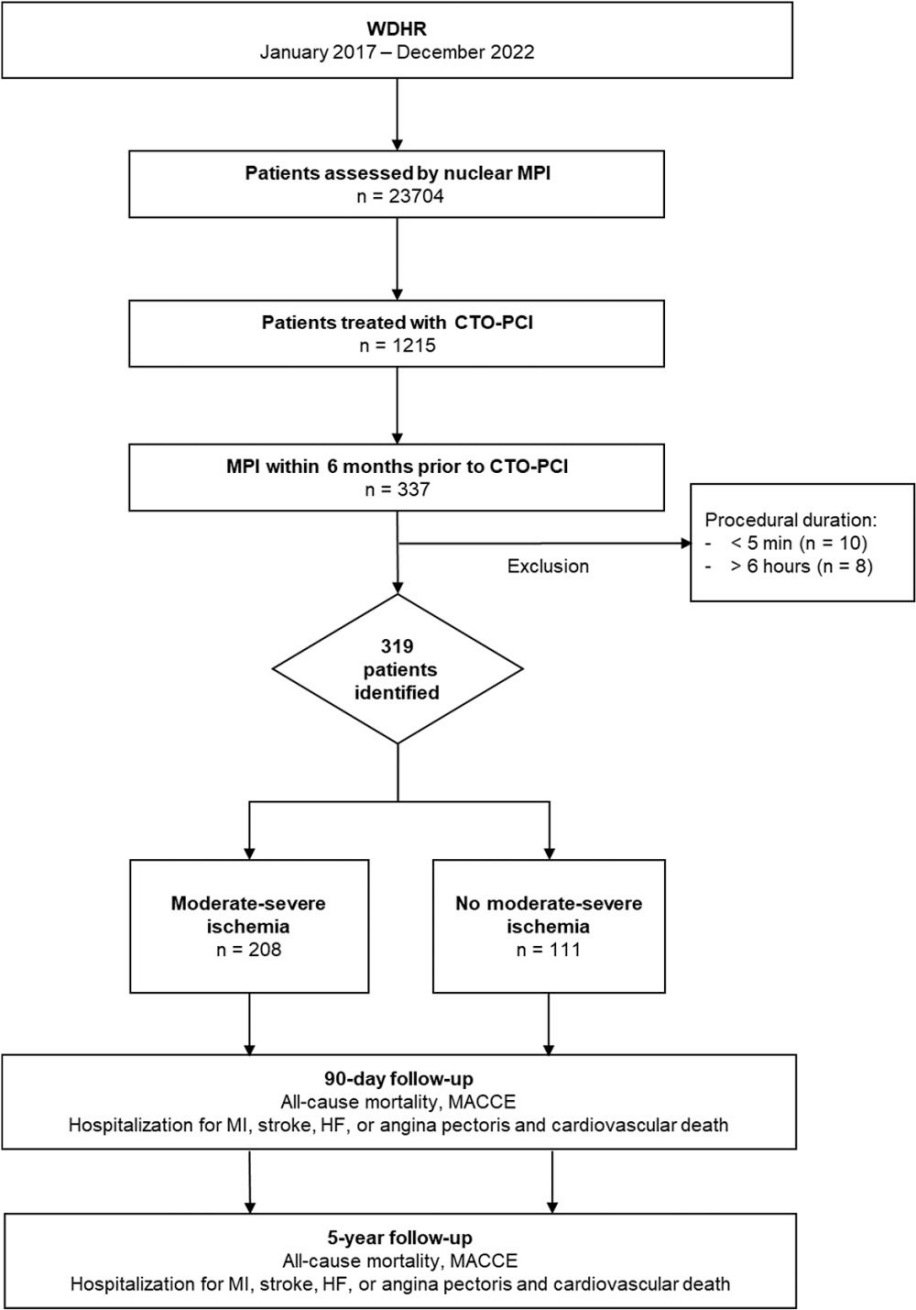
Identification and stratification diagram.

All patients registered in WDHR who had been assessed by MPI with SPECT or PET and subsequently treated with CTO-PCI within 6 months during the period between January 2017 and December 2022 were included (*Figure 1*). The Capital Region of Denmark authorized data access in accordance with the General Data Protection Regulation. Under Danish law, ethics committee approval and informed consent are not required for registry-based studies conducted for statistical or scientific research purposes.

MPI results were evaluated by a nuclear medicine physician based on semiquantitative and quantitative assessment of perfusion abnormalities and ischaemia and subsequently registered in the WDHR. Stress testing was performed using intravenous adenosine or regadenoson or by dynamic exercise on a bicycle ergometer, depending on local instructions. Attenuation correction was applied as standard practice across all sites. Registered balanced ischaemia was interpreted as potential multivessel or left main stenosis.

CTOs were identified by invasive coronary angiography, and CTO-PCI was performed at high-volume centres by a dedicated CTO operator using drug-eluting stents (DES) or alternatively plain old balloon angioplasty (POBA) in unsuccessful attempts. PCI data were prospectively registered in the WDHR by the operator, with a binary indicator variable denoting a CTO-PCI. Patient information on sex and date of birth was retrieved from the Danish Civil Registration System.^29^ Patient comorbidities and anatomical and procedural characteristics were obtained from WDHR at the time of PCI. Patient data on previous MI and CABG as well as outcome-defining diagnoses were retrieved from the National Danish Patient Registry.^30^ Patient medication data were retrieved from the Danish National Prescription Registry. Medical treatment was defined as at least one redeemed prescription within 90 days before or after PCI using anatomical therapeutic chemical codes (see Supplementary data online, *Table S1*).

### Exclusion and stratification

The following exclusion criteria were applied: (i) invasive procedural duration of less than 5 min, (ii) invasive procedural duration exceeding 6 h, as these extremes were considered potential registration errors, and (iii) patient migration between MPI and CTO-PCI.

Based on MPI evaluations, patients were stratified into two groups:

- Moderate–severe ischaemia: Patients presenting with ≥10% ischaemia of the myocardium or balanced ischaemia, with or without MI sequelae.
- No moderate–severe ischaemia: Patients with normal perfusion or abnormal perfusion defined as one of the following:
  1. MI sequelae alone.
  2. Ischaemia in <10% of the myocardium, with or without MI sequelae.

### Outcomes

Outcomes were retrieved from the Danish National Patient Registry.^30^ The co-primary outcomes were (i) all-cause mortality and (ii) a composite major adverse cardio- and cerebrovascular events (MACCE) comprising cardiovascular death, MI, hospitalization for stroke, hospitalization for heart failure (HF), and hospitalization for angina pectoris. Secondary outcomes were individual components of MACCE: (i) cardiovascular death, (ii) MI, (iii) stroke, (iv) hospitalization for HF, and (v) hospitalization for angina pectoris.

Hospitalization was defined as a hospital admission lasting more than 12 h. Diagnoses were classified according to International Statistical Classification of Diseases 9th and 10th revision (ICD-8 and ICD-10) (see Supplementary data online, *Table S2*). Patients were followed continuously from the date of the CTO-PCI procedure until the occurrence of an event, censoring, or a maximum follow-up of 90 days and 5 years. Patients were censored due to migration, competing events, lost to follow-up, or reaching the maximum follow-up time.

A subgroup analysis was performed including only patients assessed by PET-MPI. Reporting followed the guidelines of the RECORD statement.^31^

### Statistical analysis

Continuous variables are presented as mean ± standard deviation (SD) or as medians with first and third interquartile ranges (IQR), while categorical variables are reported as frequencies and percentages. Comparisons of continuous variables were conducted using *t*-tests, and comparisons of categorical variables were performed with χ^2^ test. Kaplan–Meier curves were generated for all-cause mortality at 90 days and 5 years. The Aalen–Johansen estimator was used to construct cumulative incidence curves for MACCE, cardiovascular death and hospitalization due to MI, stroke, HF, and angina pectoris at 90 days and 5 years, accounting for non-cardiovascular death as a competing risk for MACCE and cardiovascular death and all-cause mortality as a competing risk for MI, stroke, HF, and angina pectoris. Groups were compared using univariable and multivariable models to determine outcome-specific unadjusted (uHR) and adjusted hazard ratios (aHR) with 95% confidence intervals (CI). Cox regression analysis was employed for all-cause mortality and competing risk regression according to the method of Fine and Gray was used for outcomes subject to competing risk. Multivariable models adjusted for patient age, sex, diabetes, previous CABG, and previous MI. Median follow-up times were estimated using the reverse Kaplan–Meier method. All tests were performed two-sided, and statistical significance was set at *P* < 0.05. Patients with missing data were excluded from respective analyses. All analyses were conducted using R (version 4.4.1) within RStudio 2023.03.2, Posit Software, Boston, MA, USA.

## Results

### Demographics

A total of 319 patients evaluated by MPI and subsequently treated with CTO-PCI within 6 months were identified. Ten patients were excluded due to short procedural duration, and eight were excluded due to long procedural duration. The mean age of the cohort was 68 years (SD 10.1 years) with 254 of 319 (79.6%) being male. In the population, 91 of 319 (30.7%) had diabetes, and 70 of 319 (21.9%) were previously treated with CABG. In total, 208 of 319 (65.2%) were identified with moderate–severe ischaemia, while 111 of 319 (34.8%) patients were not. While procedural success was achieved in 275 of 319 (86.2%) of the population, and 5 of 208 (2.4%) of patients with moderate–severe ischaemia were treated with POBA as compared to 10 of 111 (9.0%) patients without moderate–severe ischaemia (*P* = 0.029). All patients who received stents were treated with DES. Overall, patient and procedural characteristics were comparable between the two groups (see *Tables 1 and 2*), except for the MPI modality, with 160 of 208 (76.9%) of patients with moderate–severe ischaemia having undergone PET compared with 52 of 111 (46.8%) of patients without moderate–severe ischaemia (*P* < 0.001). Among patients assessed by PET, significantly more patients with moderate–severe ischaemia had been assessed by [^15^O]H_2_O-PET and [^13^N]NH_3_-PET [63 of 208 (39%) vs. 6 of 111 (11.5%), *P* < 0.001]. Among patients assessed by SPECT, all patients were assessed by ^99m^Tc sestamibi-SPECT or ^99m^Tc-tetrofosmin-SPECT. Patients with moderate–severe ischaemia had lower Summed Rest Score [3.3 (SD 5.2) vs. 4.7 (SD 6), *P* = 0.029] indicating a lower level of permanent perfusion defects and were more often in treatment with nitrates [124 of 208 (59.6% vs. 49) of 111 (44.1%), *P* = 0.01].

**Table 1:** Patient characteristics

Variable	Level	Moderate–severe ischaemia (*n* = 208)	No moderate–severe ischaemia (*n* = 111)	Total (*n* = 319)	*P*-value
**Age**	Mean (SD)	67.9 (10.3)	68.2 (9.8)	68 (10.1)	0.81
**Sex**	Male	168 (80.8)	86 (77.5)	254 (79.6)	0.58
**Family history of IHD**	*n* (%)	79 (42.0)	37 (42.5)	116 (42.2)	1
	Missing	20	24	44	
**Smoking**	Former moker	88 (46.8)	50 (54.3)	138 (49.3)	0.47
	Never smoker	60 (31.9)	24 (26.1)	84 (30.0)	
	Smoker	40 (21.3)	18 (19.6)	58 (20.7)	
	Missing	20	19	39	
**Diabetes**	*n* (%)	62 (32.1)	29 (28.2)	91 (30.7)	0.57
	Missing	15	8	23	
**Hypercholesterolemia**	*n* (%)	175 (86.6)	87 (85.3)	262 (86.2)	0.89
	Missing	6	9	15	
**Hypertension**	*n* (%)	161 (78.5)	75 (73.5)	236 (76.9)	0.40
	Missing	3	9	12	
**Acetylsalicylic acid**	*n* (%)	175 (84.1)	96 (86.5)	271 (85.0)	0.69
**Statin**	*n* (%)	197 (94.7)	106 (95.5)	303 (95.0)	0.97
**P2Y_12_-inhibitors**	*n* (%)	196 (94.2)	103 (92.8)	299 (93.7)	0.79
**Nitrates**	*n* (%)	84 (40.4)	62 (55.9)	146 (45.8)	0.011
**Beta-blockers**	*n* (%)	165 (79.3)	87 (78.4)	252 (79.0)	0.96
**Calcium channel blockers**	*n* (%)	106 (51.0)	48 (43.2)	154 (48.3)	0.23
**Diuretics**	*n* (%)	93 (44.7)	58 (52.3)	151 (47.3)	0.24
**RAAS-inhibitors**	*n* (%)	131 (63.0)	77 (69.4)	208 (65.2)	0.31
**Previous CABG**	*n* (%)	45 (21.6)	24 (21.6)	69 (21.6)	1
**Previous MI**	*n* (%)	73 (35.1)	44 (39.6)	117 (36.7)	0.5
**Previous PCI**	*n* (%)	77 (37.4)	45 (42.1)	122 (39.0)	0.49
	Missing	2	4	6	
**Revascularization between MPI and CTO-PCI**	*n* (%)	4 (1.9)	7 (6.3)	11 (3.4)	0.09
**LVEF**	Mean (SD)	49.7 (11.5)	48.4 (11.7)	49.2 (11.6)	0.35
	Missing	25	8	33	
**Body mass index**	Mean (SD)	29 (4.1)	28.4 (4.3)	28.8 (4.2)	0.25
	Missing	15	1	16	
**Body surface area**	Mean (SD)	2 (0.2)	2 (0.2)	2 (0.2)	0.16
	Missing	15	1	16	
**Creatinine clearance**	Mean (SD)	92.4 (39.5)	85.2 (29.6)	89.9 (36.5)	0.13
	Missing	32	19	51	

IHD, ischaemic heart disease; RAAS, renin–angiotensin–aldosterone system

**Table 2:** Myocardial perfusion imaging and procedural characteristics

Variable	Level	Moderate–severe ischaemia (*n* = 208)	No moderate–severe ischaemia (*n* = 111)	Total (*n* = 319)	*P*-value
**MPI result**	MI sequelae and ischaemia, > 10%	64 (30.8)	0 (0.0)	64 (20.1)	
	No abnormal perfusion	0 (0.0)	66 (59.5)	66 (20.7)	
	Ischaemia, > 10%	135 (64.9)	0 (0.0)	135 (42.3)	
	MI sequelae	0 (0.0)	10 (9.0)	10 (3.1)	
	Ischaemia, ≤ 10%	0 (0.0)	8 (7.2)	8 (2.5)	
	Balanced ischaemia	9 (4.3)	0 (0.0)	9 (2.8)	
	MI sequelae and ischaemia, ≤ 10%	0 (0.0)	5 (4.5)	5 (1.6)	
	Other^b^	0 (0.0)	22 (19.8)	22 (6.9)	
**MPI Modality** **− MPI Tracer**	PET − ^82^Rubidium − [^15^O]H_2_O or [^13^N]NH_3_	160 (76.9) 97 (60.6) 63 (39.4)	52 (46.8) 46 (88.5) 6 (11.5)	212 (66.5) 143 (67.5) 69 (32.5)	<0.001^c^ <0.001^d^
	SPECT^§^	48 (23.1)	59 (53.2)	107 (33.5)	
**SRS**	Mean (SD)	3.3 (5.2)	4.7 (6)	3.8 (5.6)	0.029
	Missing	1	0	1	
**SSS**	Mean (SD)	10 (10.1)	10.8 (8.8)	10.3 (9.6)	0.48
	Missing	1	0	1	
**SDS**	Mean (SD)	6.6 (7)	5.6 (6)	6.3 (6.7)	0.19
	Missing	1	0	1	
**Rest perfusion**	Mean (SD)	1 (0.3)	1 (0.2)	1 (0.3)	0.36
	Missing	51	63	114	
**Stress perfusion**	Mean (SD)	1.8 (0.6)	1.8 (0.6)	1.8 (0.6)	0.84
	Missing	51	65	116	
**Coronary flow reserve**	Mean (SD)	1.9 (0.7)	1.9 (0.7)	1.9 (0.7)	0.82
	Missing	51	65	116	
**MPI indication**	Angina/Anginal equivalent	173 (83.2)	96 (86.5)	269 (84.3)	0.72
	Heart failure	20 (9.6)	8 (7.2)	28 (8.8)	
	Other clinical indication	15 (7.2)	7 (6.3)	22 (6.9)	
**CTO-PCI of LM or LAD**	*n* (%)	62 (29.8)	30 (27.0)	92 (28.8)	0.69
**CTO-PCI of RCA**	*n* (%)	122 (58.7)	55 (49.5)	177 (55.5)	0.15
**CTO-PCI of CX**	*n* (%)	31 (14.9)	27 (24.3)	58 (18.2)	0.05
**PCI indication**	Stable angina pectoris or documented ischaemia	189 (90.9)	92 (82.9)	281 (88.1)	0.06
	Other	19 (9.1)	19 (17.1)	38 (11.9)	
**Intervention type**	Stent	170 (81.7)	85 (76.6)	255 (79.9)	0.029
	POBA or other	5 (2.4)	10 (9.0)	15 (4.7)	
	Failed	33 (15.9)	16 (14.4)	49 (15.4)	
**Number of stents**	Mean (SD)	2.5 (1.2)	2.5 (1.2)	2.5 (1.2)	0.94
	Missing	38	26	64	
**Length of stent**	Mean (SD)	78 (36)	73.9 (37.7)	76.6 (36.6)	0.40
	Missing	38	26	64	
**TIMI-flow after intervention**	3	173 (83.2)	88 (79.3)	261 (81.8)	0.22
	1–2	5 (2.4)	7 (6.3)	12 (3.8)	
	0	30 (14.4)	16 (14.4)	46 (14.4)	
**Coronary arteries treated**	1	160 (76.9)	83 (74.8)	243 (76.2)	0.77
	>1	48 (23.1)	28 (25.2)	76 (23.8)	
**Lesions treated**	1	153 (73.6)	72 (64.9)	225 (70.5)	0.05
	>1	51 (24.5)	39 (35.1)	90 (28.2)	
	0	4 (1.9)	0 (0.0)	4 (1.3)	
**Combined CTO-PCI and regular PCI**	Yes	52 (25.0)	38 (34.2)	90 (28.2)	0.11
**Degree of revascularization**	Complete	143 (68.8)	78 (70.3)	221 (69.3)	0.88
	Incomplete	65 (31.2)	33 (29.7)	98 (30.7)	
**Procedural success**	Yes	179 (86.1)	96 (86.5)	275 (86.2)	1
**Periprocedural complications^a^**	Yes	12 (5.8)	5 (4.5)	17 (5.3)	0.83

LM, left main; LAD, left anterior descending artery; RCA, right coronary artery; CX, left circumflex artery; POBA, plain old ballon angioplasty; SRS, summed rest score; SSS, summed stress score; SDS, summed difference score.

^a^Includes arrythmia, pace requirement, defibrillation, contrast reaction, catheter-induced vascular injuries, respiratory insufficiency, cardiogenic shock, cardiac tamponade, acute percutaneous transluminal coronary angioplasty, acute CABG, stroke, cardiac arrest, vasopressor requirement.

^b^MPI result not otherwise covered by the listed result types. ^§99m^Tc sestamibi-SPECT or ^99m^Tc tetrofosmin-SPECT.

^c^Comparing overall use of PET vs. SPECT between groups.

^d^Compared use of tracer within PET group.

### Outcomes

**Figure 2:**
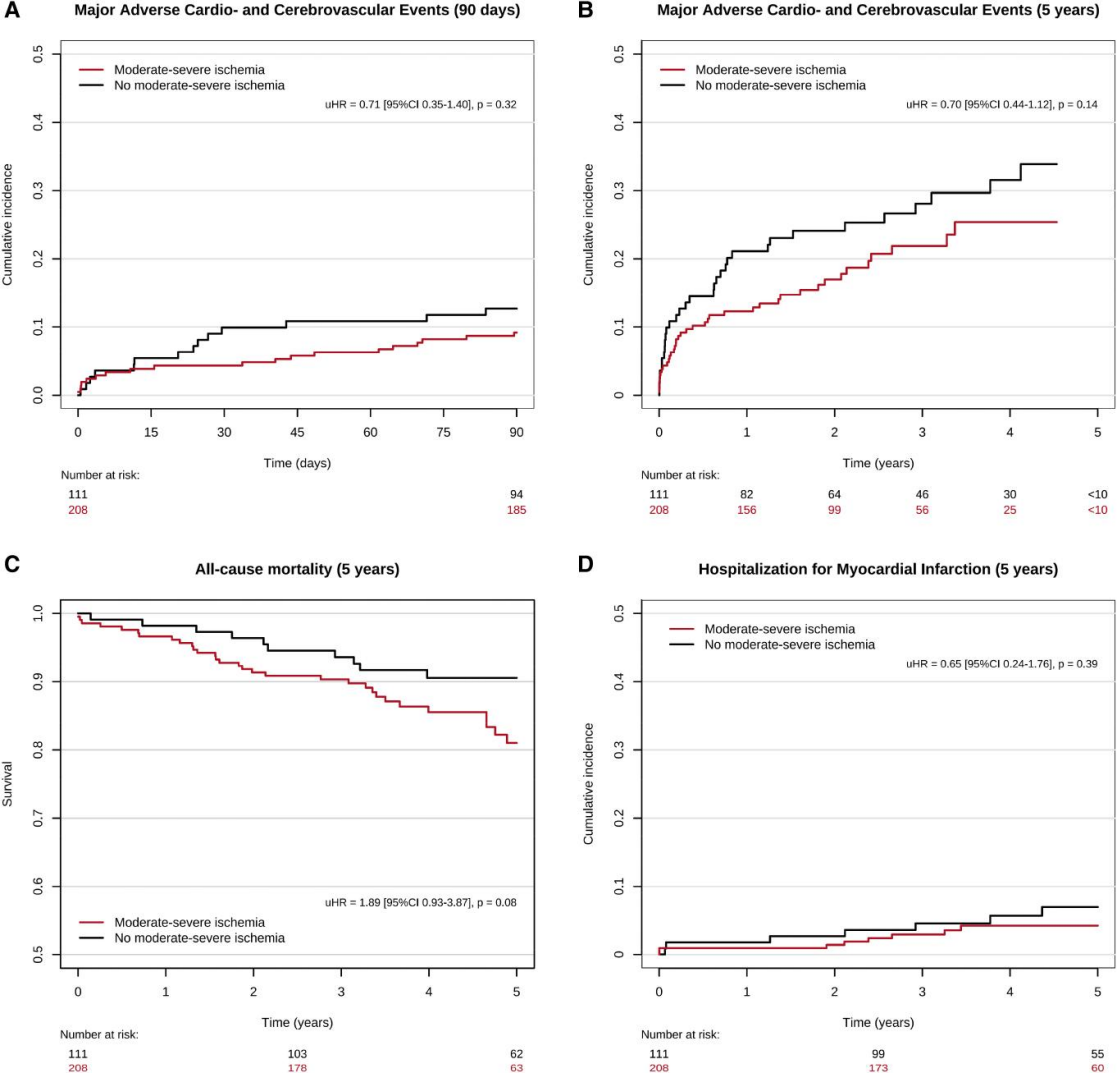
Cumulative incidence curves of *A*) Major adverse cardio- and cerebrovascular events over 90 days and *B*) 5 years, *C*) myocardial infarction over 5 years and Kaplan–Meier curves for *D*) all-cause mortality over 5 years following percutaneous coronary intervention for chronic total occlusion (CTO-PCI). Y-axes are cut at 0.5. Red: Moderate–severe ischaemia. Black: No moderate–severe ischaemia.

**Figure 3:**
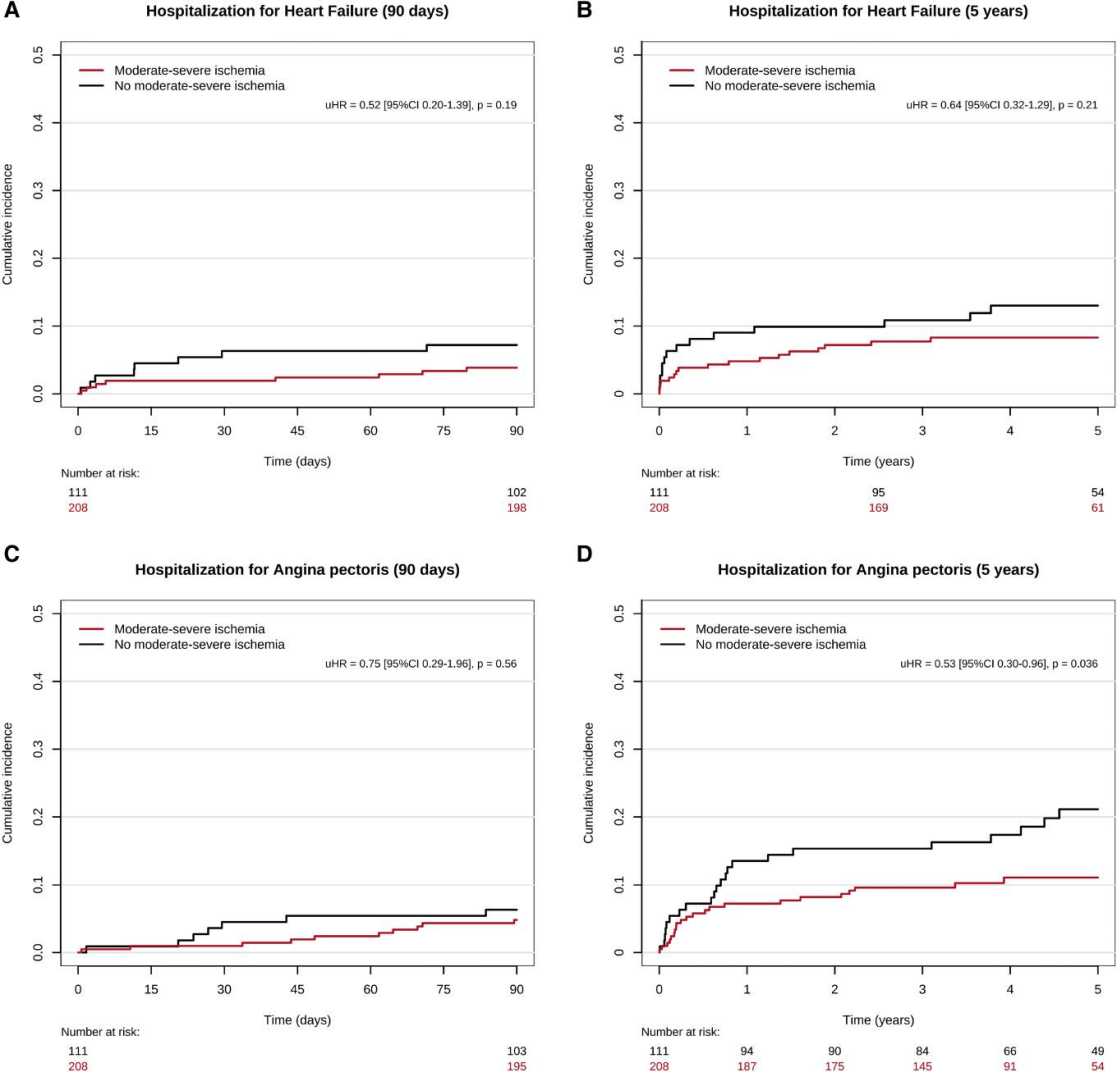
Cumulative incidence curves heart failure of *A*) 90 days and *B*) 5 years and angina pectoris over *C*) 90 days and *D*) 5 years following percutaneous coronary intervention for chronic total occlusion (CTO-PCI). Y-axes are cut at 0.5. Red: Moderate–severe ischaemia. Black: No moderate–severe ischaemia.

**Figure 4:**
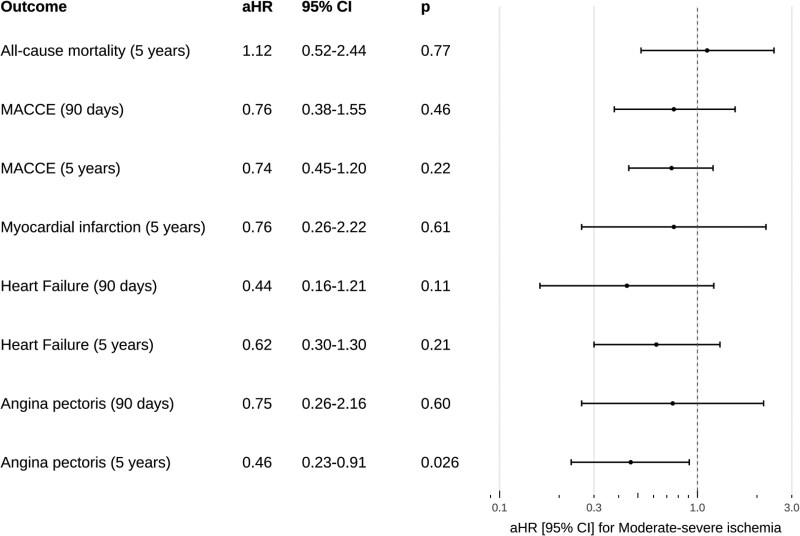
Forest plot of aHR and 95% CI for patients with moderate–severe ischaemia compared with patients without moderate–severe ischaemia in each outcome analysis following percutaneous coronary intervention for chronic total occlusion (CTO-PCI).

Cumulative incidence curves and Kaplan–Meier curves and corresponding unadjusted hazard ratios are depicted in *Figures 2* and *3*. A summary of time-to-event estimates and median follow-up times are provided in Supplementary data online, *Table S3*. A summary of adjusted hazard ratios for all outcomes is provided in *Figure 4*.

#### All-cause mortality

Median follow-up time was 3.76 years (IQR 3.25–5.00 years). At 5-year follow-up, 22 of 208 patients with moderate–severe ischaemia and 10 of 111 patients without moderate–severe ischaemia had died, yielding an estimated survival probability was 86.34% (95% CI 80.55–92.55%) vs. 89.54% (95% CI 83.52–96.00%) for each group, respectively. Univariable Cox regression found no difference in mortality between patients with or without moderate–severe ischaemia [uHR 1.37 (95% CI 0.65–2.90), *P* = 0.41]. Multivariable Cox regression found age as a predictor of all-cause mortality at 5 years (see Supplementary data online, *Table S4*). Adjusting for confounding factors, no difference was found between patients with and without moderate–severe ischaemia [aHR 1.12 (95% CI 0.52–2.43), *P* = 0.77].

#### Major adverse cardio- and cerebrovascular events

Median follow-up time was 2.69 years (IQR 0.88–3.38 years). Twenty of 208 patients with moderate–severe ischaemia and 14 of 111 patients without moderate–severe ischaemia experienced MACCE at 90-day follow-up, corresponding to a cumulative incidence of MACCE of 9.67% (95% CI 5.64–13.69%) and 12.69% (95% CI 6.46–18.87%), respectively. At 5 years, 45 of 208 and 33 of 111 had experienced MACCE in each group, resulting in a cumulative incidence of 29.28% (95% CI 20.98–37.57%) vs. 35.64% (95% CI 25.00–46.27%), respectively. Univariable competing risk regression showed no difference in the risk of MACCE in patients with moderate–severe ischaemia at both 90 days [uHR 0.75 (95% CI 0.38–1.47), *P* = 0.40] and 5 years [uHR 0.80 (95% CI 0.51–1.25), *P* = 0.32]. No significant predictors were identified in multivariable models (see Supplementary material 4). After adjusting for confounding factors, no difference remained in the risk of MACCE at 90 days [aHR 0.76 (95% CI 0.38–1.55), *P* = 0.46] and 5 years [aHR 0.74 (95% CI 0.45–1.20), *P* = 0.22].

#### Secondary outcomes

Median follow-up time was 3.76 years (IQR 3.17–5.00 years) for hospitalization for MI. At 5 years, 8 of 208 and 7 of 111 of patients with and without moderate–severe ischaemia were hospitalized for MI, resulting in a cumulative incidence of 5.31% (95% CI 1.59–9.03%) vs. 7.85% (95% CI 2.08–13.63%) for each group, respectively. Univariable competing risk regression found no difference between groups [uHR 0.72 (95% CI 0.26–1.97), *P* = 0.52]. Multivariable models found previous MI as a significant predictor of MI at 5 years (see Supplementary data online, *Table S5*). Adjusting for confounding factors, no difference was found between groups [aHR 0.76 (95% CI 0.26–2.22), *P* = 0.61].

Median follow-up time was 3.89 years (IQR 3.07–5.00 years) for hospitalization for HF. At 90 days, 8 of 208 and 8 of 111 patients in each group were hospitalized for HF, corresponding to cumulative incidence of 3.85% (95% CI 1.23–6.46%) and 7.21% (95% CI 2.40–12.02%), respectively. At 5 years, 17 of 208 and 14 of 111 in each group were hospitalized for HF, yielding to a cumulative incidence of 8.82% (95% CI 4.75–12.89%) and 13.61% (95% CI 6.86–20.35%). Univariable competing risk regression found no difference between groups at 90 days [uHR 0.52 (95% CI 0.20–1.39), *P* = 0.19] or 5 years [uHR 0.66 (95% CI 0.33–1.34), *P* = 0.25]. Multivariable models identified age as a significant predictor of HF at 5 years (see Supplementary data online, *Table S5*). After adjustment, no difference in risk of hospitalization for HF was found at 90 days [aHR 0.44 (95% CI 0.16–1.21), *P* = 0.11] or 5 years [aHR 0.62 (95% CI 0.30–1.30), *P* = 0.21].

Median follow-up time was 3.71 years (IQR 2.68–5 years) for hospitalization for angina pectoris. At 90 days, 10 of 208 and 7 of 111 in each group were hospitalized for angina pectoris, yielding a cumulative incidence of 4.81% (95% CI 1.90–7.71%) vs. 6.31% (95% CI 1.78–10.83%), for each group. At 5 years, 21 of 208 and 22 of 111 were hospitalized for angina pectoris, representing a cumulative incidence of 11.55% (95% CI 6.61–16.50%) vs. 22.63% (95% CI 13.94–31.33%) for each group, respectively. Univariable competing risk regression found no difference between groups at 90 days [uHR 0.75 (95% CI 0.29–1.96), *P* = 0.56], but a statistically significant decreased risk in patients with moderate–severe ischaemia at 5 years [uHR 0.53 (95% CI 0.29–0.96), *P* = 0.038]. Multivariable models identified no significant predictors at either follow-up time (see Supplementary data online, *Table S5*). After adjustment for confounding factors, no difference in risk of hospitalization for angina pectoris was found in between groups at 90 days [aHR 0.75 (95% CI 0.26–2.16), *P* = 0.60] but a significant difference at 5 years [aHR 0.46 (95% CI 0.23–0.91), *P* = 0.026].

#### Subgroup analysis

Results from the subgroup analyses containing patients assessed by PET are depicted in Supplementary data online, *Tables S6*–*9* and *Figures S1 and S2*. Among patients assessed by PET (*n* = 212) patients with moderate–severe ischaemia had higher body mass index and a higher prevalence of hypertension and treatment with calcium channel blockers. A higher proportion of patients without moderate–severe ischaemia were treated with a combined CTO-PCI and regular PCI during the same procedure. No differences were found between patients with and without moderate–severe ischaemia in outcome analyses including all-cause mortality, MACCE, hospitalization for HF, or angina.

## Discussion

This study evaluated the association between non-invasively assessed moderate–severe myocardial ischaemia and clinical outcomes in unselected patients undergoing CTO-PCI in a regionwide registry. The main result showed that the presence of moderate–severe ischaemia was not associated with differences in all-cause mortality or hospitalization for MI. However, a non-significant trend towards decreased risk of MACCE and hospitalization for HF was observed in patients with moderate–severe ischaemia. The risk of hospitalization for angina pectoris was comparable between groups at 90 days, and a small difference was observed between groups at 5 years, suggesting that patients with moderate–severe ischaemia have a decreased long-term risk of hospitalization for angina pectoris compared with those without moderate–severe ischaemia after CTO-PCI. A higher ischaemic burden may indicate patients more likely to achieve symptomatic relief following CTO-PCI compared with those with a lower ischaemic burden. These findings are consistent with current guidelines, which highlight ischaemic symptom improvement as the primary indication for CTO-PCI.^1^ However, proper patient reported quality of life and standardized angina scoring instruments should be employed to better determine this effect on symptomatic relief. Hospitalization for angina does not exactly reflect the general symptoms of the population but is rather an estimate of the proportion of patients with symptoms severe enough to require hospitalization, e.g. unstable angina or crescendo angina. Likewise, the results of the current study are limited to the outcome following CTO-PCI after the treatment decision; further studies comparing CTO-PCI to optimal medical treatment are needed to determine the treatment decision itself.

### The role of MPI in the evaluation of CTO

Both SPECT and PET MPI have been used in the current literature to select patients for CTO-PCI and to assess outcomes and physiological parameters following the procedure. CTO-PCI has been shown to increase myocardial perfusion and decrease end-diastolic volume as measured by SPECT.^32^ Studies by Ma *et al*. and Wright *et al*. further suggest that CTO-induced ischaemia detected by SPECT can accurately predict the risk of adverse events in unrevascularized patients, indicating that MPI may aid in risk stratification of patients for whom revascularization is considered.^33,34^ In recent years, the use of SPECT MPI has declined, while PET has seen a gradual increase in utilization.^35^ A meta-analysis by An *et al*. on the evaluation of patients with CTOs using PET found that CTO-PCI increased stress myocardial blood flow and coronary flow reserve.^36^ This improved perfusion was observed both in the CTO region and in remote myocardial areas after CTO-PCI, which the authors’ suggested could be attributable to collateral circulation. Furthermore, PET-detected reductions in ischaemic burden after CTO-PCI are associated with decreased mortality, lower rates of MI, and normalization of myocardial blood flow with long-term relief from cardiac symptoms.^37,38^ By incorporating both imaging modalities, Safley *et al*. identified a significant decrease in ischaemia on SPECT or PET following CTO-PCI, with an associated improvement in long-term survival.^39^ Contrary to the results of this study, these prior studies suggest a potential role for MPI in evaluating CTO-PCI with respect to both physiological parameters and hard cardiac outcomes. The ongoing ISCHEMIA-CTO trial (NCT03563417) randomizes patients with evidence of myocardial ischaemia assessed by PET, SPECT, cardiac magnetic resonance imaging, or dobutamine stress echocardiography to medical therapy vs. CTO-PCI to evaluate safety, clinical outcomes, and symptomatic improvement.^40^

### Limitations

This study has several limitations. The stratification of patients in this study based on both SPECT and PET MPI should be interpreted with caution due to the differences in accuracy between the two MPI modalities: In stable ischaemic heart disease, using fractional flow reserve as a reference, SPECT shows a sensitivity of no more than 70% and specificity around 80%, while PET achieves a sensitivity of 90% and specificity of 85%.^41–43^ As SPECT has lower sensitivity compared with PET, the observed differences in MPI modality between groups may suggest the presence of undetected moderate–severe ischaemia in the SPECT group, a non-differential misclassification biasing the differences between the groups towards the null. A similar misclassification bias may be found among patients assessed by PET, where a larger proportion of patients allocated to the group of moderate–severe ischaemia had been assessed by [^15^O]H_2_O-PET or [^13^N]NH_3_-PET. While [^15^O]H_2_O-PET is generally considered the reference among PET-tracers for MPI,^44^ current literature describes a similar diagnostic accuracy to ^82^Rubidium-PET.^45,46^ Another potential source of misclassification bias is the term balanced ischaemia, which for this study was interpreted as potential multivessel or left main disease but remains an uncertain term depending on the interpretation of the registering physician.^47,48^ The results from the performed subgroup analysis containing patients assessed by PET were overall limited by a small sample size as well as differences in patient and procedural characteristics between groups; results from this subgroup analysis should be interpreted with caution.

As for all registry-based studies, data quality is inherently dependent on the accuracy of the input. Consequently, errors and missing data may be present, which should be considered when interpreting the results. Moreover, any positive result can only be hypothesis generating.

While procedural success was overall comparable between groups, outcomes may have been influenced by differences in intervention, as more patients without moderate–severe ischaemia were treated with POBA. Additionally, the short follow-up times for long-term analyses and the high number of censored observations prior to 5 years of follow-up represent a limitation. These issues are largely attributable to the relatively recent inclusion of MPI data in the WDHR, as noted by Søby *et al*.: Data collection began in 2017, but reporting from all centres was first implemented in 2019.^28^ As a result, several patients did not complete the full 5-year follow-up, reducing the number at risk in later follow-up periods, decreasing the precision of the estimates, and leading to wider confidence intervals. Another limitation of this study is that models did not account for potential time-dependent effects, which is suggested by the crossing cumulative incidence curves indicating a possible violation of the proportional hazards assumption. More studies with larger cohorts, extended follow-up, and randomization to MPI are needed to accurately evaluate the role of MPI in the pre-procedural assessment of CTO-PCI patients.

## Conclusion

In patients assessed by nuclear MPI prior to treatment with CTO-PCI the presence of moderate–severe ischaemia was not associated with differences in all-cause mortality, MACCE, hospitalization for MI, or HF compared with patients without moderate–severe ischaemia. However, patients with moderate–severe ischaemia exhibited a lower long-term risk of hospitalization for angina pectoris. Given the observational nature of this study, randomized trials are needed to validate these results.

## Supplementary Material

qyaf137_Supplementary_Data

## Data Availability

Data used in this study were obtained from Danish health registries and are available only through permission from the Danish health authorities.

## Lead author biography

Jesper Boes Henningsen is a highly talented medical student who has worked as a research assistant at Department of Cardiology, Aalborg University Hospital, and currently at Department of Cardiothoracic Ssurgery, Aalborg University Hospital.
