# Supplementary material for: Impact of myocardial perfusion abnormalities on clinical outcomes in patients treated with percutaneous coronary intervention for chronic total occlusions
Source: Eur Heart J Imaging Methods Pract. 2026 Jan 8;4(1):qyaf137. doi: 10.1093/ehjimp/qyaf137 (PMC12781094; doi:10.1093/ehjimp/qyaf137)
Supplement: qyaf137_Supplementary_Data [file qyaf137_supplementary_data.docx]

Supplementary material

Contents

[Supplementary table 1: Definitions of medication according to ATC-codes. 2](#_Toc203480833)

[Supplementary table 2: Definitions of outcome diagnoses according to ICD-10 and ICD-8 codes. 2](#_Toc203480834)

[Supplementary table 3: Time-to-event results. 3](#_Toc203480835)

[Supplementary table 4: Multivariable regression results for primary outcome analyses. 4](#_Toc203480836)

[Supplementary table 5: Multivariable regression results for secondary outcome analyses 5](#_Toc203480837)

[Supplementary table 6: Patient characteristics for subgroup-analysis including PET-MPI. 6](#_Toc203480838)

[Supplementary table 7: Procedural characteristics for subgroup-analysis including PET-MPI. 7](#_Toc203480839)

[Supplementary table 8: Multivariable regression results for primary outcome analyses in subgroup-analysis including PET-MPI. 8](#_Toc203480840)

[Supplementary table 9: Multivariable regression results for secondary outcome analyses in subgroup-analysis including PET-MPI. 9](#_Toc203480841)

[Supplementary figure 1: Time-to-event analysis of primary outcomes for subgroup including patients assessed by PET-MPI. 10](#_Toc203480842)

[Supplementary figure 2: Time-to-event analysis of secondary outcomes for subgroup including patients assessed by PET-MPI. 11](#_Toc203480844)

Supplementary table 1: Definitions of medication according to ATC-codes.

| Medication | ATC codes |
| --- | --- |
| Statin | C10 |
| Acetylsalicylic acid | B01AC06 |
| P2Y12-inhibitors | B01AC06, B01AC22, B01AC24 |
| Nitrates | C01DA |
| Diuretics | C03 |
| Calcium channel blockers | C08 |
| Betablockers | C07 |
| RAAS-inhibitors | C09 |

Supplementary table 2: Definitions of outcome diagnoses according to ICD-10 and ICD-8 codes.

| Outcome diagnosis | ICD-10 codes | ICD-9 codes |
| --- | --- | --- |
| Myocardial infarction | I21.xx | 410 |
| Angina pectoris | I20.xx | 413 |
| Stroke | DI63, DI64, DG458, DG459 | 433, 434, 435, 436 |
| Heart failure | DI110, DI50 | 428 |
| Cardiovascular death | Death attributed to I00-99 | Death attributed to 390-458 |

Supplementary table 3: Time-to-event results. For each outcome analysis cumulative incidence or survival probability (for all-cause mortality) with 95% confidence interval (CI) and median follow-up times.

| Outcome | Maximum follow-up time | Moderate-severe ischemia | Cumulative incidence [95% CI] or  Survival probability [95% CI] | Median follow-up time (IQR) |
| --- | --- | --- | --- | --- |
| MACCE | 90 days | Yes | 9.67% [5.64%-13.69%] | 2,69 years  (0.88–3.38 years) |
|  |  | No | 12.67% [6.46%-18.87%] |  |
|  | 5 years | Yes | 29.28% [20.98%-37.57%] |  |
|  |  | No | 35.64% [25.00%-46.27%] |  |
| All-cause mortality | 5 years | Yes | 86.34% [80.55%-92.55%] | 3,76 years  (3.25–5.00 years) |
|  |  | No | 89.54% [83.52%-96.00%] |  |
| Myocardial Infarction | 5 years | Yes | 5.31% [1.59%-9.03%] | 3,76 years  (3.17-5.00 years) |
|  |  | No | 7.85% [2.08%-13.63%] |  |
| Hospitalization for Heart Failure | 90 days | Yes | 3.85% [1.23%-6.46%] | 3,85 years  (3.07-5.00 years) |
|  |  | No | 7.21% [2.40%-12.02%] |  |
|  | 5 years | Yes | 8.82% [4.75%-12.89%] |  |
|  |  | No | 13.61% [6.86%-20.35%] |  |
| Hospitalization for Angina pectoris | 90 days | Yes | 4.81% [1.90%-7.71%] | 3,71 years  (2.68-5 years) |
|  |  | No | 6.31% [1.78%-10.83%] |  |
|  | 5 years | Yes | 11.55% [6.61%-16.50%] |  |
|  |  | No | 22.63% [13.94%-31.33%] |  |

Supplementary table 4: Multivariable regression results for primary outcome analyses. Adjusted hazard ratios (aHR) with 95% confidence intervals (95% CI) and p-values for each covariate in each multivariable model. Major Adverse Cardio- and Cerebrovascular Events were calculated using competing risk regression while all-cause mortality was calculated using Cox regression. Abbreviations: CABG: Coronary Artery Bypass Graft; MACCE: Major Adverse Cardio- and Cerebrovascular Events; MI: Myocardial Infarction. * = p < 0,05.

| Outcome  (Maximum follow-up time) | Covariate | aHR [95% CI] | p-value |
| --- | --- | --- | --- |
| MACCE  (90 days) | Moderate-severe ischemia | 0.76 [0.38-1.55] | 0.46 |
|  | Age | 1.02 [0.98-1.06] | 0.33 |
|  | Sex = Male | 0.66 [0.30-1.44] | 0.30 |
|  | Diabetes | 1.04 [0.48-2.24] | 0.92 |
|  | Previous CABG | 1.04 [0.43-2.54] | 0.93 |
|  | Previous MI | 0.81 [0.36-1.81] | 0.61 |
| MACCE  (5 years) | Moderate-severe ischemia | 0.74 [0.45-1.20] | 0.22 |
|  | Age | 1.03 [1.00-1.06] | 0.08 |
|  | Sex = Male | 1.10 [0.59-2.06] | 0.76 |
|  | Diabetes | 1.36 [0.81-2.27] | 0.24 |
|  | Previous CABG | 0.97 [0.55-1.71] | 0.90 |
|  | Previous MI | 0.93 [0.55-1.56] | 0.78 |
| All-cause mortality  (5 years) | Moderate-severe ischemia | 1.12 [0.52-2.44] | 0.77 |
|  | Age | 1.15 [1.09-1.21] | <0.001 |
|  | Sex = Male | 0.94 [0.40-2.25] | 0.90 |
|  | Diabetes | 2.79 [1.32-5.90] | 0.007 |
|  | Previous CABG | 0.95 [0.39-2.29] | 0.90 |
|  | Previous MI | 0.92 [0.42-2.02] | 0.84 |

Supplementary table 5: Multivariable regression results for secondary outcome analyses**.** Adjusted hazard ratios (aHR) with 95% confidence intervals (95% CI) and p-value for each covariate in multivariable competing risk regression. Abbreviations: CABG: Coronary Artery Bypass Graft; MI: Myocardial Infarction. * = p < 0,05.

| Outcome  (Maximum follow-up time) | Covariate | aHR [95% CI] | p-value |
| --- | --- | --- | --- |
| Myocardial Infarction  (5 years) | Moderate-severe ischemia | 0.76 [0.26- 2.22] | 0.61 |
|  | Age | 0.97 [0.92-1.02] | 0.19 |
|  | Sex = Male | 2.53 [0.32-20.29] | 0.38 |
|  | Diabetes | 0.95 [0.29- 3.09] | 0.93 |
|  | Previous CABG | 1.29 [0.44- 3.78] | 0.64 |
|  | Previous MI | 3.98 [1.34-11.81] | 0.013* |
| Hospitalization for Heart Failure  (90 days) | Moderate-severe ischemia | 0.44 [0.16-1.21] | 0.11 |
|  | Age | 1.04 [0.97-1.11] | 0.30 |
|  | Sex = Male | 1.06 [0.28-3.96] | 0.94 |
|  | Diabetes | 0.92 [0.28-3.01] | 0.89 |
|  | Previous CABG | 1.39 [0.39-4.94] | 0.61 |
|  | Previous MI | 0.58 [0.17-2.03] | 0.39 |
| Hospitalization for Heart Failure  (5 years) | Moderate-severe ischemia | 0.62 [0.30-1.30] | 0.21 |
|  | Age | 1.05 [1.00-1.10] | 0.038* |
|  | Sex = Male | 1.86 [0.62-5.55] | 0.27 |
|  | Diabetes | 1.23 [0.55-2.75] | 0.62 |
|  | Previous CABG | 0.89 [0.34-2.28] | 0.80 |
|  | Previous MI | 0.56 [0.23-1.36] | 0.20 |
| Hospitalization for Angina pectoris  (90 days) | Moderate-severe ischemia | 0.75 [0.26-2.16] | 0.60 |
|  | Age | 1.03 [0.98-1.07] | 0.23 |
|  | Sex = Male | 0.76 [0.25-2.28] | 0.62 |
|  | Diabetes | 1.61 [0.56-4.63] | 0.38 |
|  | Previous CABG | 0.97 [0.28-3.31] | 0.96 |
|  | Previous MI | 0.62 [0.20-1.98] | 0.42 |
| Hospitalization for Angina pectoris  (5 years) | Moderate-severe ischemia | 0.46 [0.23-0.91] | 0.026* |
|  | Age | 1.00 [0.96-1.03] | 0.78 |
|  | Sex = Male | 0.80 [0.38-1.70] | 0.57 |
|  | Diabetes | 1.78 [0.89-3.54] | 0.10 |
|  | Previous CABG | 1.01 [0.48-2.13] | 0.99 |
|  | Previous MI | 1.02 [0.51-2.06] | 0.95 |

Supplementary table 6: Patient characteristics for subgroup-analysis including PET-MPI. Abbreviations: SD: Standard Deviation; IHD: Ischemic Heart Disease; CABG: Coronary Artery Bypass Graft; PCI: Percutaneous Coronary Intervention; MI: Myocardial infarction. * = p < 0.05.

| Variable | Level | Moderate-severe ischemia (n=160) | No moderate-severe ischemia (n=52) | Total (n=212) | p-value |
| --- | --- | --- | --- | --- | --- |
| Age | Mean (SD) | 68.2 (10.4) | 68.1 (8.7) | 68.2 (10) | 0.93 |
| Sex | Male | 132 (82.5) | 45 (86.5) | 177 (83.5) | 0.64 |
| Family history of IHD | Yes | 60 (42.3) | 11 (32.4) | 71 (40.3) | 0.39 |
|  | missing | 18 | 18 | 36 |  |
| Smoking | Former smoker | 67 (46.5) | 21 (60.0) | 88 (49.2) | 0.33 |
|  | Never smoker | 44 (30.6) | 7 (20.0) | 51 (28.5) |  |
|  | Smoker | 33 (22.9) | 7 (20.0) | 40 (22.3) |  |
|  | missing | 16 | 17 | 33 |  |
| Diabetes | Yes | 49 (33.8) | 11 (25.0) | 60 (31.7) | 0.36 |
|  | missing | 15 | 8 | 23 |  |
| Hypercholesterolemia | Yes | 133 (86.4) | 37 (86.0) | 170 (86.3) | 1 |
|  | missing | 6 | 9 | 15 |  |
| Hypertension | Yes | 122 (77.7) | 26 (59.1) | 148 (73.6) | 0.022* |
|  | missing | 3 | 8 | 11 |  |
| Acetylsalicylic acid | Yes | 135 (84.4) | 45 (86.5) | 180 (84.9) | 0.88 |
| Statin | Yes | >144 (>90%) | >47 (>90%) | >191 (>90%) |  |
| P2Y_12_ inhibitors | Yes | >144 (>90%) | >47 (>90%) | >191 (90%) |  |
| Nitrates | Yes | 94 (58.8) | 21 (40.4) | 115 (54.2) | 0.032* |
| Betablockers | Yes | 129 (80.6) | 40 (76.9) | 169 (79.7) | 0.71 |
| Calcium channel blockers | Yes | 87 (54.4) | 14 (26.9) | 101 (47.6) | 0.001* |
| Diuretics | Yes | 76 (47.5) | 26 (50.0) | 102 (48.1) | 0.88 |
| RAAS inhibitor | Yes | 103 (64.4) | 33 (63.5) | 136 (64.2) | 1 |
| Previous CABG | Yes | 37 (23.1) | 13 (25.0) | 50 (23.6) | 0.93 |
| Previous MI | Yes | 55 (34.4) | 22 (42.3) | 77 (36.3) | 0.39 |
| Previous PCI | Yes | 57 (36.1) | 23 (47.9) | 80 (38.8) | 0.19 |
|  | missing | 2 | 4 | 6 |  |
| Revascularization between MPI and CTO-PCI | Yes | <16 (<10%) | <5 (<10%) | <21 (<10%) |  |
| Ejection fraction | Mean (SD) | 48.6 (12.1) | 46.5 (11.6) | 48.1 (12) | 0.29 |
|  | missing | 24 | 3 | 27 |  |
| Body Mass Index | Mean (SD) | 28.8 (4.3) | 27.4 (3.6) | 28.4 (4.2) | 0.043* |
|  | missing | 15 | 1 | 16 |  |
| Body Surface Area | Mean (SD) | 2 (0.2) | 2 (0.2) | 2 (0.2) | 0.26 |
|  | missing | 15 | 1 | 16 |  |
| Creatinine clearance | Mean (SD) | 90.2 (40.5) | 87.4 (29.9) | 89.5 (38.1) | 0.67 |
|  | missing | 24 | 7 | 31 |  |

Supplementary table 7: Procedural characteristics for subgroup-analysis including PET-MPI. Abbreviations MPI: Myocardial Perfusion Imaging; MI: Myocardial infarction; CTO: Chronic Total Occlusion. PCI: Percutaneous Coronary Intervention; PET: Positron Emission Tomography; CTO: Chronic Total Occlusion; LM: Left Main; LAD: Left Anterior Descending artery; RCA: Right Coronary Artery; CX: Left Circumflex artery; POBA: Plain Old Ballon Angioplasty; SRS: Summed Rest Score; SSS: Summed Stress Score; SDS: Summed Difference Score; TIMI: Thrombolysis In Myocardial Infarction. NA: Not available. * = p < 0.05. †Compared within PET group. ‡Includes arrythmia, pace requirement, defibrillation, contrast reaction, catheter induced vascular injuries, respiratory insufficiency, cardiogenic shock, cardiac tamponade, acute percutaneous transluminal coronary angioplasty, acute CABG, stroke, cardiac arrest, vasopressor requirement.

| Variable | Level | Moderate-severe ischemia (n=160) | No moderate-severe ischemia (n=52) | Total (n=212) | p-value |
| --- | --- | --- | --- | --- | --- |
| MPI result | MI sequelae and ischemia, >10% | 51 (31.9) | 0 (0.0) | 51 (24.1) |  |
|  | No abnormal perfusion | 0 (0.0) | 38 (73.1) | 38 (17.9) |  |
|  | Ischemia, >10% | 100 (62.5) | 0 (0.0) | 100 (47.2) |  |
|  | Other | 0 (0.0) | 14 (26.9) | 14 (6.6) |  |
|  | Balanced ischemia | 9 (5.6) | 0 (0.0) | 9 (4.2) |  |
| MPI modality  - MPI tracer | PET  - ^82^Rubidium  - [^15^O]H_2_O or [^13^N]NH_3_ | 160 (100.0) | 52 (100.0) | 212 (100.0) | <0.001^†^ |
|  |  | 97 (60.6) | 46 (88.5) | 143 (67.5) |  |
|  |  | 63 (39.4) | 6 (11.5) | 69 (32.5) |  |
| SRS | Mean (SD) | 3.9 (5) | 2.9 (5.3) | 3.1 (5.2) | 0.22 |
|  | Missing | 0 | 1 | 1 |  |
| SSS | Mean (SD) | 10.3 (9.9) | 8.9 (10.7) | 9.3 (10.5) | 0.42 |
|  | Missing | 0 | 1 | 1 |  |
| SDS | Mean (SD) | 6.2 (6.9) | 6.1 (7.4) | 6.2 (7.3) | 0.93 |
|  | Missing | 0 | 1 | 1 |  |
| Rest perfusion | Mean (SD) | 1 (0.2) | 1 (0.3) | 1 (0.3) | 0.36 |
|  | Missing | 4 | 3 | 7 |  |
| Stress perfusion | Mean (SD) | 1.8 (0.6) | 1.8 (0.6) | 1.8 (0.6) | 0.84 |
|  | Missing | 6 | 3 | 9 |  |
| Coronary Flow Reserve | Mean (SD) | 1.9 (0.7) | 1.9 (0.7) | 1.9 (0.7) | 0.82 |
|  | Missing | 6 | 3 | 9 |  |
| MPI indication | Angina/Anginal equivalent | 45 (86.5) | 130 (81.2) | 175 (82.5) |  |
|  | Other clinical indication | 7 (13.5) | 30 (18.8) | 37 (17.5) | 0.51 |
| CTO-PCI on LM or LAD | Yes | 50 (31.2) | 13 (25.0) | 63 (29.7) | 0.5 |
| CTO-PCI on RCA | Yes | 95 (59.4) | 29 (55.8) | 124 (58.5) | 0.77 |
| CTO-PCI on CX | Yes | 21 (13.1) | 11 (21.2) | 32 (15.1) | 0.24 |
| PCI indication | Stable angina or documented ischemia | 142 (88.8) | 40 (76.9) | 182 (85.8) | 0.06 |
|  | Other | 18 (11.2) | 12 (23.1) | 30 (14.2) |  |
| Intervention type | Stent | 44 (84.6) | 134 (83.8) | 178 (84.0) | 1 |
|  | Failed, POBA or other | 8 (15.4) | 26 (16.2) | 34 (16.0) |  |
| Number of stents | Mean (SD) | 2.9 (1.4) | 2.6 (1.2) | 2.7 (1.2) | 0.26 |
|  | Missing | 8 | 26 | 34 |  |
| Length of stent | Mean (SD) | 86.1 (43.2) | 82.8 (36.7) | 83.6 (38.3) | 0.62 |
|  | Missing | 8 | 26 | 34 |  |
| TIMI-flow after intervention | 3 | 43 (82.7) | 135 (84.4) | 178 (84.0) | 0.94 |
|  | <3 | 9 (17.3) | 25 (15.6) | 34 (16.0) |  |
| Coronary arteries treated | 1 | 123 (76.9) | 35 (67.3) | 158 (74.5) | 0.23 |
|  | >1 | 37 (23.1) | 17 (32.7) | 54 (25.5) |  |
| Lesions treated |  | NA | NA | NA |  |
| Combined CTO-PCI and regular PCI | Yes | 40 (25.0) | 22 (42.3) | 62 (29.2) | 0.027* |
| Degree of revascularization | Complete | 110 (68.8) | 40 (76.9) | 150 (70.8) | 0.34 |
|  | Incomplete | 50 (31.2) | 12 (23.1) | 62 (29.2) |  |
| Procedural success | Yes | 141 (88.1) | 48 (92.3) | 189 (89.2) | 0.56 |
| Periprocedural complications^‡^ | Yes | 11 (6.9) | 4 (7.7) | 15 (7.1) | 1 |

Supplementary table 8: Multivariable regression results for primary outcome analyses in subgroup-analysis including PET-MPI. Adjusted hazard ratios (aHR) with 95% confidence intervals (95% CI) and p-values for each covariate in each multivariable model. Major Adverse Cardio- and Cerebrovascular Events were calculated using competing risk regression while all-cause mortality was calculated using Cox regression. Abbreviations: CABG: Coronary Artery Bypass Graft; MACCE: Major Adverse Cardio- and Cerebrovascular Events; MI: Myocardial Infarction. * = p < 0,05.

| Outcome  (Maximum follow-up time) | Covariate | aHR [95% CI] | p-value |
| --- | --- | --- | --- |
| MACCE  (90 days) | Moderate-severe ischemia | 0.79 [0.33-1.88] | 0.59 |
|  | Age | 1.01 [0.97-1.05] | 0.66 |
|  | Sex = Male | 0.57 [0.21-1.52] | 0.26 |
|  | Diabetes | 0.75 [0.30-1.86] | 0.53 |
|  | Previous CABG | 1.21 [0.50-2.95] | 0.67 |
|  | Previous MI | 0.82 [0.35-1.95] | 0.65 |
| MACCE  (5 years) | Moderate-severe ischemia | 0.84 [0.45-1.56] | 0.58 |
|  | Age | 1.01 [0.98-1.04] | 0.46 |
|  | Sex = Male | 0.87 [0.38-1.99] | 0.74 |
|  | Diabetes | 0.92 [0.50-1.69] | 0.78 |
|  | Previous CABG | 1.17 [0.64-2.13] | 0.61 |
|  | Previous MI | 1.11 [0.62-2.00] | 0.73 |
| All-cause mortality  (5 years) | Moderate-severe ischemia | 1.08 [0.39-3.03] | 0.88 |
|  | Age | 1.13 [1.07-1.20] | <0.001* |
|  | Sex = Male | 0.61 [0.23-1.59] | 0.31 |
|  | Diabetes | 1.86 [0.80-4.32] | 0.15 |
|  | Previous CABG | 0.85 [0.33-2.24] | 0.75 |
|  | Previous MI | 0.79 [0.32-1.95] | 0.61 |

Supplementary table 9: Multivariable regression results for secondary outcome analyses in subgroup-analysis including PET-MPI. Adjusted hazard ratios (aHR) with 95% confidence intervals (95% CI) and p-value for each covariate in multivariable competing risk regression. Abbreviations: CABG: Coronary Artery Bypass Graft; MI: Myocardial Infarction. * = p < 0,05.

| Outcome  (Maximum follow-up time) | Covariate | aHR [95% CI] | p-value |
| --- | --- | --- | --- |
| Hospitalization for Heart Failure  (90 days) | Moderate-severe ischemia | 0.34 [0.11-1.03] | 0.06 |
|  | Age | 1.01 [0.94-1.08] | 0.75 |
|  | Sex = Male | 0.81 [0.16-4.25] | 0.81 |
|  | Diabetes | 0.72 [0.18-2.91] | 0.65 |
|  | Previous CABG | 1.41 [0.42-4.73] | 0.58 |
|  | Previous MI | 0.53 [0.14-2.03] | 0.36 |
| Hospitalization for Heart Failure  (5 years) | Moderate-severe ischemia | 0.52 [0.22-1.22] | 0.14 |
|  | Age | 1.03 [0.98-1.08] | 0.21 |
|  | Sex = Male | 1.38 [0.38-5.02] | 0.63 |
|  | Diabetes | 0.99 [0.40-2.46] | 0.98 |
|  | Previous CABG | 0.87 [0.34-2.24] | 0.77 |
|  | Previous MI | 0.61 [0.24-1.55] | 0.30 |
| Hospitalization for Angina pectoris  (5 years) | Moderate-severe ischemia | 0.60 [0.24-1.49] | 0.27 |
|  | Age | 0.97 [0.93-1.01] | 0.18 |
|  | Sex = Male | 0.72 [0.24-2.13] | 0.55 |
|  | Diabetes | 1.18 [0.46-3.00] | 0.73 |
|  | Previous CABG | 1.18 [0.50-2.82] | 0.71 |
|  | Previous MI | 1.37 [0.59-3.15] | 0.46 |

Supplementary figure 1: Time-to-event analysis of primary outcomes for subgroup including patients assessed by PET-MPI. Cumulative incidence curves for Major Adverse Cardio- and Cerebrovascular Events over 90 days and 5 years and Kaplan-Meier curves for All-cause mortality over 5 years following percutaneous coronary intervention for chronic total occlusion (CTO-PCI). Y-axes are cut at 0.5. Red: Moderate-severe ischemia. Black: No moderate-severe ischemia. Abbreviations: uHR: Unadjusted Hazard Ratio between groups; CI: Confidence interval.


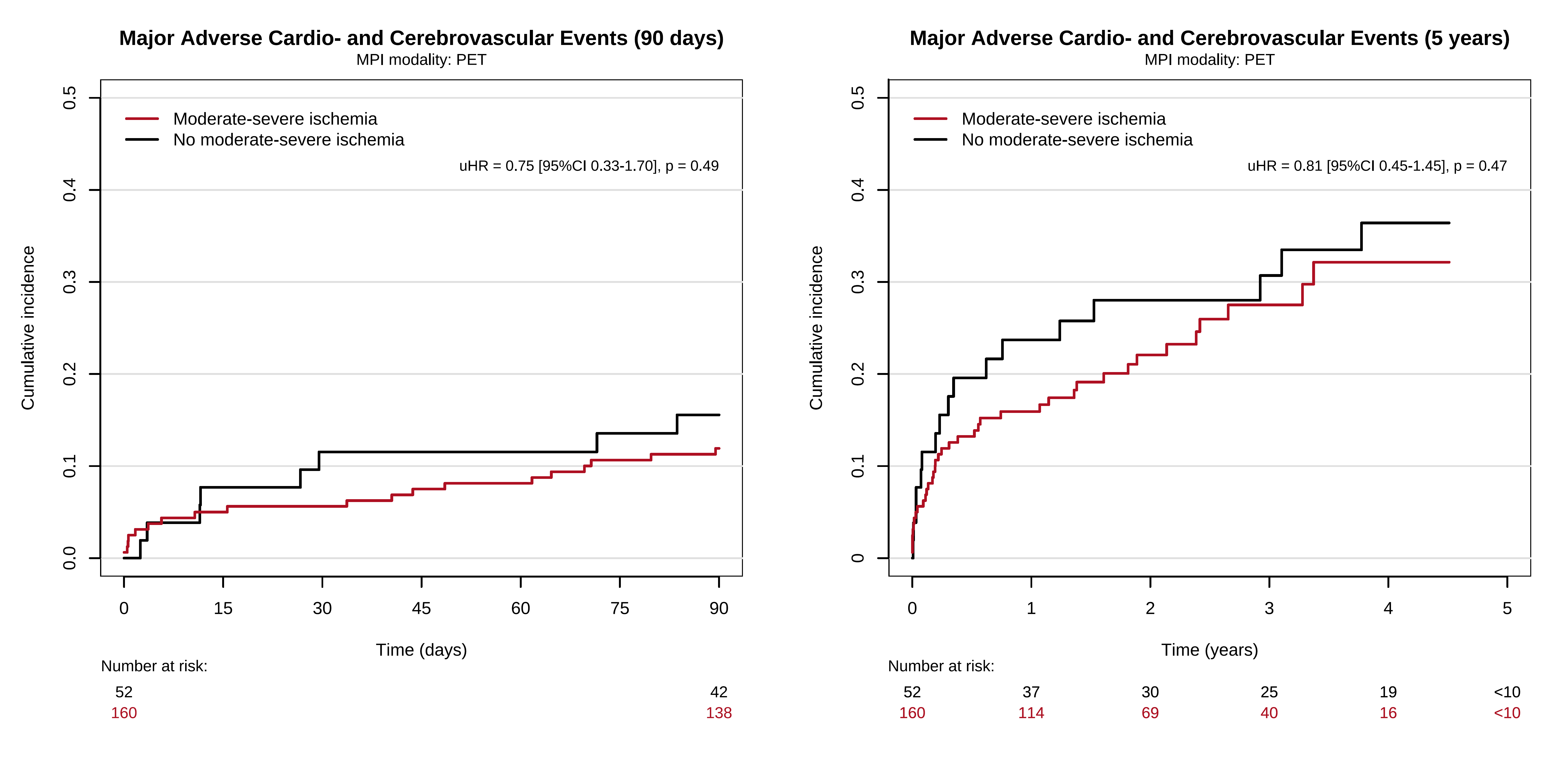


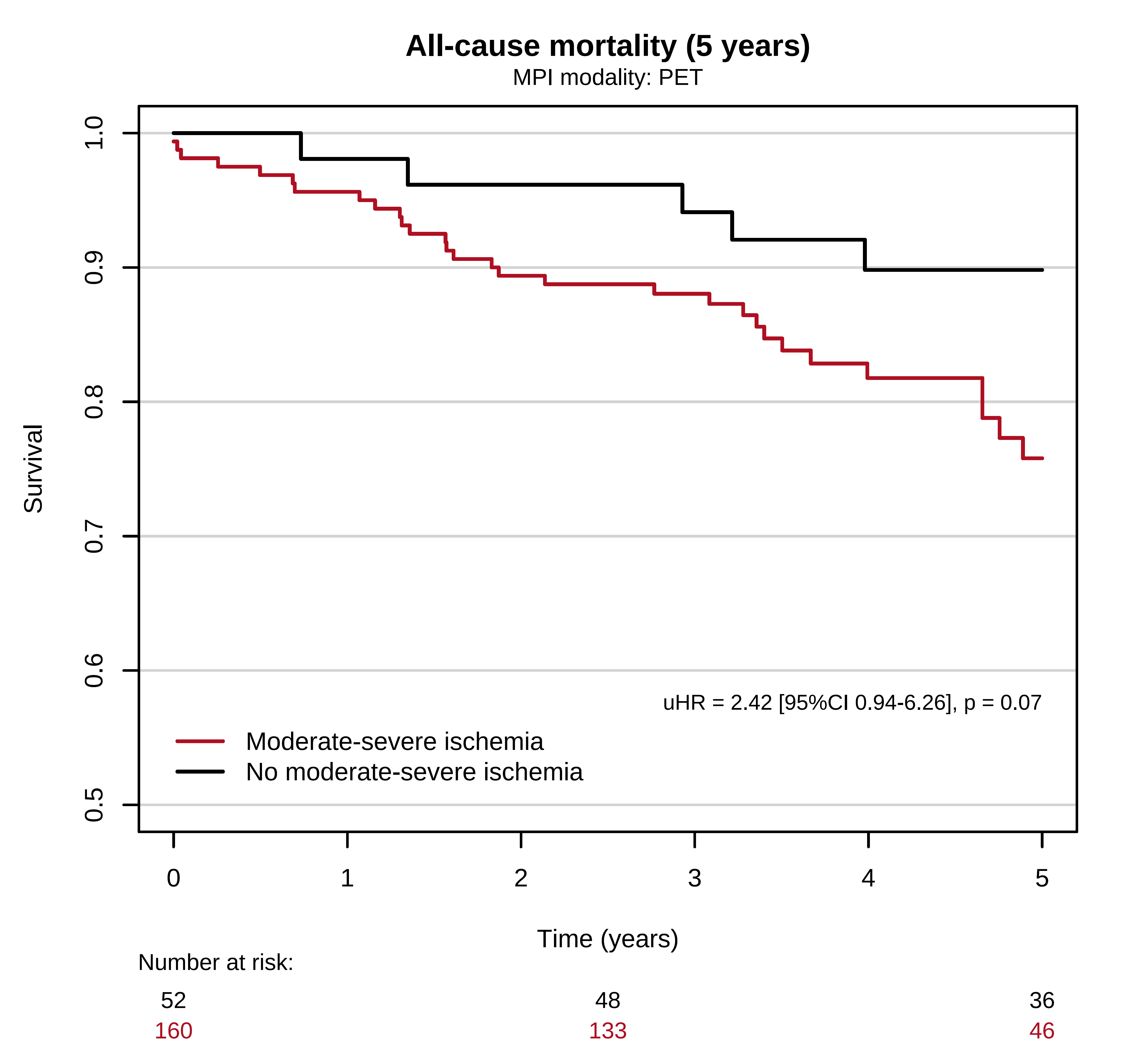


Supplementary figure 2: Time-to-event analysis of secondary outcomes for subgroup including patients assessed by PET-MPI. Cumulative incidence curves heart failure over 90 days and 5 years and angina pectoris over 5 years following percutaneous coronary intervention for chronic total occlusion (CTO-PCI). Y-axes are cut at 0.5. Red: Moderate-severe ischemia. Black: No moderate-severe ischemia. Abbreviations: uHR: Unadjusted Hazard Ratio between groups; CI: Confidence interval.
